# O_2_ partitioning of sulfur oxidizing bacteria drives acidity and thiosulfate distributions in mining waters

**DOI:** 10.1038/s41467-023-37426-8

**Published:** 2023-04-10

**Authors:** Kelly J. Whaley-Martin, Lin-Xing Chen, Tara Colenbrander Nelson, Jennifer Gordon, Rose Kantor, Lauren E. Twible, Stephanie Marshall, Sam McGarry, Laura Rossi, Benoit Bessette, Christian Baron, Simon Apte, Jillian F. Banfield, Lesley A. Warren

**Affiliations:** 1 University of Toronto, Toronto, ON Canada; 2 Environmental Resources management (ERM), Toronto, ON Canada; 3 Department of Earth and Planetary Science, University of California, Berkeley, CA USA; 4 McMaster University, Hamilton, ON Canada; 5 Glencore, Sudbury Integrated Nickel Operations, Sudbury, ON Canada; 6 Université de Montréal, Montréal, Montréal, QC Canada; 7 CSIRO Land and Water, Clayton, NSW Australia

**Keywords:** Element cycles, Environmental chemistry, Water microbiology, Gene expression profiling

## Abstract

The acidification of water in mining areas is a global environmental issue primarily catalyzed by sulfur-oxidizing bacteria (SOB). Little is known about microbial sulfur cycling in circumneutral pH mine tailing impoundment waters. Here we investigate biological sulfur oxidation over four years in a mine tailings impoundment water cap, integrating aqueous sulfur geochemistry, genome-resolved metagenomics and metatranscriptomics. The microbial community is consistently dominated by neutrophilic, chemolithoautotrophic SOB (relative abundances of ~76% in 2015, ~55% in 2016/2017 and ~60% in 2018). Results reveal two SOB strategies alternately dominate across the four years, influencing acid generation and sulfur speciation. Under oxic conditions, novel *Halothiobacillus* drive lower pH conditions (as low as 4.3) and lower [S_2_O_3_^2−^] via the complete Sox pathway coupled to O_2_. Under anoxic conditions, *Thiobacillus* spp. dominate in activity, via the incomplete Sox and rDSR pathways coupled to NO_3_^−^, resulting in higher [S_2_O_3_^2−^] and no net significant acidity generation. This study provides genomic evidence explaining acidity generation and thiosulfate accumulation patterns in a circumneutral mine tailing impoundment and has significant environmental applications in preventing the discharge of sulfur compounds that can impact downstream environments. These insights illuminate opportunities for in situ biotreatment of reduced sulfur compounds and prediction of acidification events using gene-based monitoring and in situ RNA detection.

## Abstract (web summary)

Microbial genomics is a widely under-utilized tool in mining in understanding water quality drivers. Here the authors show early acid generation and thiosulfate concentrations are driven by O2 dependent microbial sulfur oxidizing bacterial niches in a mine tailings impoundment

## Introduction

Most of the world’s metals come from sulfide minerals, and the produced mining wastes (mining impacted waters) and tailings (aqueous slurries of post-processed rocks) are commonly rich in highly altered and reactive sulfur compounds^1^. Microbial sulfur oxidation processes are environmentally significant as they can drive acidity generation and oxygen consumption in mining-impacted waters^1,2^. Recent reports are highlighting the global prevalence and substantial spatial scale of mine tailings facilities^3^. Significantly apparent is how limited biogeochemical knowledge of these systems is compared to the scale of socio-environmental and ecological risk that is posed if these systems fail in their purpose to mitigate impacts to local watersheds^3^. Mine tailings and wastewaters are held in retention ponds (tailings impoundments/pit lakes) on site, as they require management and treatment before the impacted waters can be discharged to receiving downstream rivers or lakes. The water cover in a tailing impoundment serves to suppress/slow the direct oxidation by oxygen of the reduced sulfur in the underlying tailings. There is an additional known advantage that in the near surface and more oxygenated portions of the water cap, dissolved reduced sulfur compounds can undergo oxidation. Thus, the associated acidification and oxygen consumption occurs in these managed on-site locations rather than further downstream in offsite receiving environments. The severe lack of knowledge of the biological controls on the sulfur chemistry of these impoundment waters creates uncertainty and risk obscuring potential mitigation strategies. Particularly, genome investigations of endemic sulfur oxidizing bacteria (SOB) and their sulfur oxidation pathways that drive sulfur biogeochemical cycling within such systems remain a critical gap. Elucidating the microbial pathways that control the oxidation of sulfur compounds, such as thiosulfate (common in sulfide ore tailings^4^), throughout mine water treatment systems will lead to improved treatment technologies. As mining activities continue to expand to meet the green technology transformation, there is a vested interest across the scientific, environmental, and mining communities to constrain biological sulfur cycling in these large-scale wastewater systems.

Microbial sulfur cycling is highly complex, involving a wide range of sulfur compounds with varying stabilities, oxidation states and interlinked microbial reactions^5,6^. Combined geochemical and “-omic” approaches have been applied to elucidate biogeochemical sulfur cycling in natural contexts including hydrothermal vents and marine settings^7–10^, as well as acid mine drainage (AMD) sites^11–13^. Mine tailing impoundments represent a further important environmental context to examine biologically driven sulfur oxidation processes due to their unique geochemical conditions including high sulfur and metal concentrations, and often high concentrations of nitrate (local rock blasting utilizes ammonium nitrate) with steep oxygen gradients. However, they have not been well studied through ‘-omic’ approaches with only limited studies to date that were based on microbial community 16S rRNA gene analyses^14–16^. Emerging results have identified novel sulfur-cycling organisms in those circumneutral mining impacted water bodies^16,17^, divergent from those found in more well studied AMD or natural sulfur rich systems^2,18–21^.

A number of sulfur oxidation genes and pathways have been identified^5^, but the distribution and environmental factors that drive their selection are not well defined. Microbial sulfur oxidation in nature is primarily carried out through the sulfur oxidation (Sox) enzymes^22^ and the reverse dissimilatory sulfite reductase (rDSR) pathways^23^, as well as the tetrathionate intermediate (S4I) pathway^24,25^. The Sox complex is well characterized and carried out by diverse taxonomic bacteria encoding *soxXYZABCD*^26–28^. The complete Sox complex produces no free reduced sulfur intermediates, as they are covalently attached to the carrier proteins SoxYZ before being fully oxidized to SO_4_^2−^^29^. However, some SOB have an incomplete Sox complex (i.e., lacking soxCD), and the generated sulfane sulfur is transported to the cytoplasm in the form of persulfides (R-S-S^−^)^30^, and further oxidized to sulfite by rDSR^23^ or sHdr (sulfur-oxidizing heterodisulfide reductase-like)^31,32^. The rDSR pathway encoded by *dsr* genes has been hypothesized to be more prevalent under anoxic conditions due its higher energy conservation efficiency^33^ and documented with wide distribution in microbial lineages^34^. The generated sulfite is further oxidized to sulfate by other enzymes, for example, from sulfite to APS (Adenosine 5’-phosphosulfate) by AprBA with the electron transferred by AprM or HdrAACB^35^, and then to sulfate by Sat^36^, or directly from sulfite to sulfate by SoeABC^37^. The intermediate tetrathionate (S4I) pathway is not particularly well constrained to date but involves the formation of free tetrathionate via the oxidation of thiosulfate by TsdA^24^ or thiosulfate:quinol oxidoreductase (TQO, encoded by DoxDA)^38^. The generated tetrathionate could be further oxidized, for example in *Acidithiobacillus*^39,40^ and *Advenella*^41^ by tetrathionate hydrolase (TetH), producing elemental sulfur, thiosulfate and sulfate. Further oxidation of the generated elemental sulfur could be performed by sulfur dioxygenase (sdo)^42^. Thus, it seems that the geochemical outcomes in nature of acidity generation and reduced sulfur compound distributions will differ for each of the Sox, rDSR and S4I pathways. This is particularly pertinent if these pathways are spatially or temporally segregated by differing environmental conditions such as oxygen levels. Autotrophic SOBs that express these pathways may also have the capacity for aerobic respiration and/or denitrification, thus O_2_, NO_3_^−^ and other intermediate N compounds are plausible terminal electron acceptors for Sox, rDSR and S4I pathways. In circumneutral S-rich environmental settings where multiple terminal electron acceptors are present (e.g., O_2_, NO_3_^−^, NO_2_^−^), SOB may utilize an array of reactions to oxidize^43,44^ and/or disproportionate sulfur compounds^45^. Environmental selective pressures (i.e. oxygen and nitrate availability) select for organisms containing the adaptive traits suitable for different conditions allowing them to outcompete their counterparts with different functional genes. We therefore hypothesize that ecological niche partitioning of SOB across spatial and temporal scales will occur in tailings impoundments.

In this study, we collected samples throughout a mine tailing impoundment water column (~38 m in depth) at an active Ni/Cu mine site over a period of four years (2015–2018). The microbial community structure, metabolic capabilities and gene transcriptional activities are examined to elucidate hypothesized trends in overarching water chemistry (pH, O_2_, sulfur and nitrogen speciation) along with water physico-chemistry, and sulfur and nitrogen speciation. Microbial members of two dominant genera (*Halothiobacillus* and *Thiobacillus*) are identified to be carrying out distinctive sulfur oxidative strategies that evidence significant connections in time and depth with acidity generation and thiosulfate concentrations (a dominant SOI species generated in sulfide ore associated tailings and an important sulfur substrate for SOB). A species of the neutrophilic, chemolithoautrophic, sulfur oxidizer *Halothiobacillus* is ubiquitous in this mining wastewater system and when significantly active, efficiently drives thiosulfate oxidation through aerobic respiration and the straight-path Sox pathway resulting in acidity generation. In contrast, when *Thiobacillus* dominates, it conducts sulfur oxidation and likely disproportionation through the incomplete Sox complex and rDSR pathways under micro-oxic or anoxic conditions, resulting in higher thiosulfate concentrations and circum-neutral pH conditions.

## Results

### Spatial and temporal trends in microbial community composition, physico-chemistry and aqueous sulfur geochemistry

**Fig. 1:**
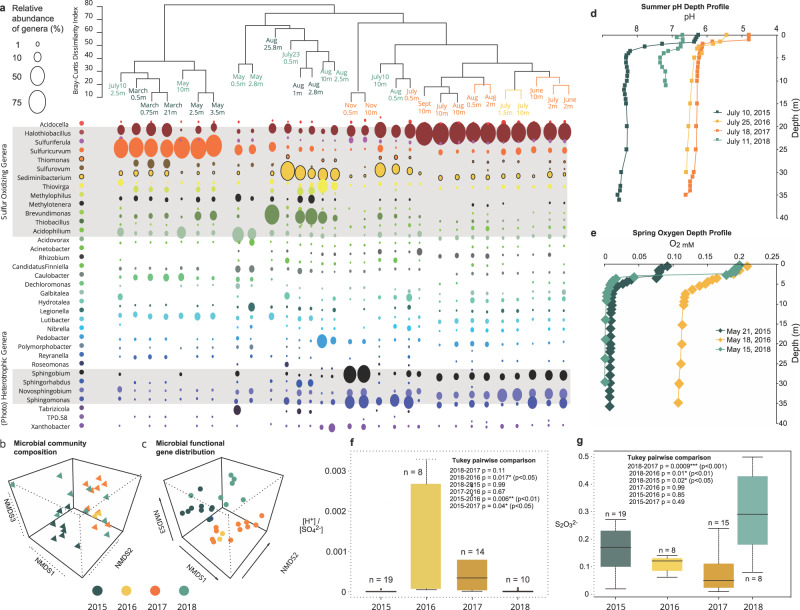
Microbial community and aqueous geochemical distributions over time. **a** Bray Curtis dissimilarity clustering of microbial communities and bubble plot representation of relative abundances of bacterial genera (showing only genera present at >1% within at least two samples). Non-metri**c** dimensional scaling (NMDS) (*k* = 3) representation of the microbial community composition **b**, and functionality (**c**) (derived as the relative abundance of sulfur cycling, nitrogen cycling, hydrogen cycling, carbon fixation, photosynthetic and aerobic respiration genes (Supplementary Dataset 5), (*n* = 30) stress = 0.065, non-metric fit = 0.996, linear fit, R^2^ = 0.97). **d** The pH depth profiles from July 2015 to 2018. **e** Spring dissolved oxygen (mM) depth profiles in 2015, 2016 and 2018. **f** Box-plot and statistical (ANOVA and subsequent Tukey pairwise statistical comparison) of annual [H^+^]/[SO_4_^2−^] values (omitting May 2017 0.5 m and May 2018 21 m where both pH and SO_4_^2−^ were unavailable). **g** Box-plot and statistical (ANOVA and subsequent Tukey pairwise statistical comparison) of annual observed [S_2_O_3_^2−^] (mM) in tailings impoundment waters inclusive of March 2015 to August 2018 with the exception of May 2018 (0.5 m), July 11, 2018 (2.8 m) July 23, 2018 (0.5 m) when data were not available. Two outliers included in the statistical (ANOVA and subsequent Tukey pairwise statistical comparison statistical comparisons were excluded from the graph for visualization purposes (March 2015 at 21 m (0.59 mM) and June 2018 at 0.5 m (1.7 mM); see Supplementary Dataset 3). In **f** and **g**, box plots enclose the first to third quartiles of data values, with a black line at the median value.

Complimentary 16S rRNA gene based and metagenomic analyses of 30 water samples collected throughout 2015–2018 from the mine tailing impoundment were characterized in tandem with water chemistry (Supplementary Dataset 1). A total of 3,533,030 16S rRNA gene sequences were obtained from 30 water samples collected throughout 2015–2018. Both 16S rRNA gene based and metagenomic analyses (Supplementary Dataset 4) indicated that these waters were inhabited by communities that varied across time in composition and diversity but were dominated by chemolithoautotrophic SOB for all 4 years (~76% in 2015, ~55% in 2016/2017 and ~60% in 2018 via 16S rRNA gene analyses). *Halothiobacillus, Sulfuricurvum*, *Thiobacillus* and *Sediminibacterium* were the most abundant genera across years but annual differences in their abundance occurred (Fig. 1a). Bray-Curtis Cluster and non-metric-dimensional scaling (NMDS) 3-dimensional analysis revealed three temporal clusters for both microbial community structure and metabolic capability (inferred by gene relative abundance (Fig. 1b, c; Supplementary Dataset 4). These three community structure clusters were driven by SOB abundance changes for *Halothiobacillus* sp. (dominance in 2016/2017) relative to higher abundances of *Sulfuricurvum*, *Thiobacillus* and *Sediminibacterium* in 2015 and 2018.

**Fig. 2:**
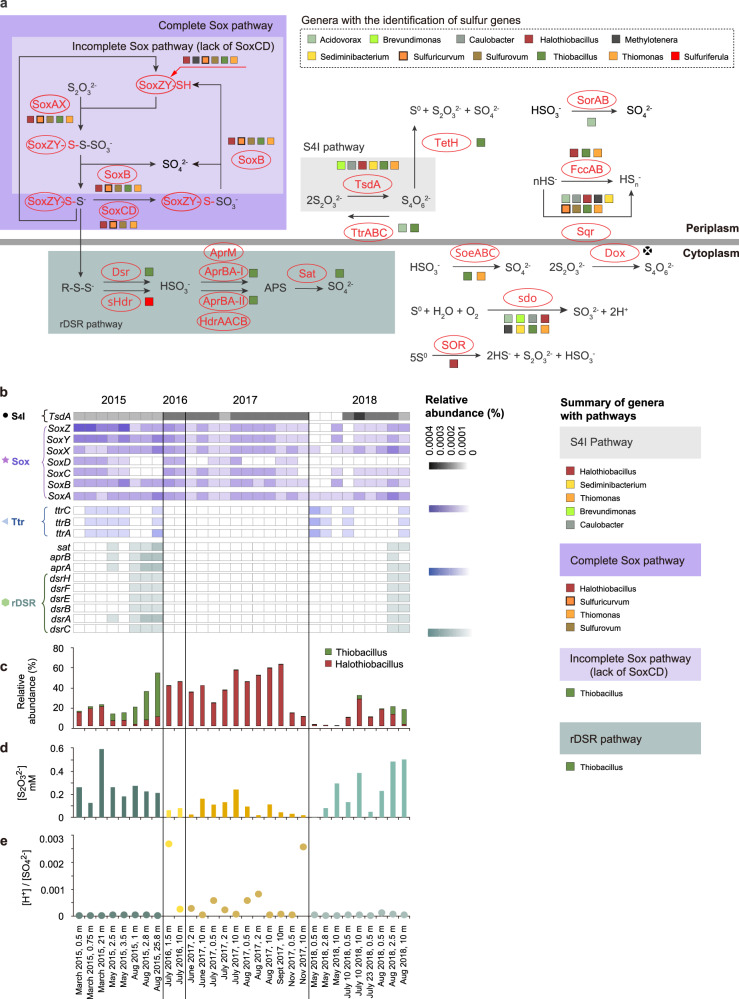
Metabolic capacity for sulfur oxidative strategies over time in the tailings impoundment/pit lake, significant sulfur oxidizers and acidity and thiosulfate outcomes. **a** Schematic of potential sulfur oxidation pathways based on identified genes encoding sulfur metabolic enzymes. A black circle with “X” indicates the gene was not detected in the genomes of the listed genera. This pathway representation was adapted from Watanabe et al.^28^. **b** Community level sulfur oxidative pathway gene distributions (*TsdA*, *soxABCDXYZ, ttrABC dsrABEFH*, a*prAB* and *sat.)*. **c** Relative abundances of *Halothiobacillus* (complete Sox pathway genes) and *Thiobacillus* (incomplete *Sox* and *rDSR* pathway genes). **d** The distribution of S_2_O_3_^2−^. **e** [H^+^]/[SO_4_^2−^] ratios from 2015 to 2018 in the tailings impoundment.

The primary chemolithoautotrophic sulfur bacterium in 2016 and 2017 was *Halothiobacillus* sp. (50 ± 21%), while *Sulfuricurvum* and *Sediminibacterium* were present at far lower abundances (total ~3%), and *Thiobacillus* was undetectable (Fig. 1a; Supplementary Dataset 4). The microbial communities of 2015 and 2018 formed their own individual clusters, as the tailing impoundment waters from 2015 (*n* = 8) were dominated by chemolithoautotrophic sulfur oxidizers *Sulfuricurvum* (37 ± 28%), *Halothiobacillus* (12 ± 8%), *Sediminibacterium* (5.9 ± 9%) and *Thiobacillus* (sulfur oxidizer and disproportionator; 17 ± 20%) (Figs. 1 and 2). In 2018, there was a similar pattern to 2015 detected with a rebound in *Sulfuricurvum* (17 ± 26%) and a re-emergence of *Thiobacillus* (3.9 ± 6%), while *Halothiobacillus* abundance decreased (11 ± 11%). However, greater diversity was observed in the microbial communities from 2018, indicated by the presence of all four of these major sulfur metabolizing groups, as well as *Thiovirga* (5 ± 8%) and *Acidovorax* (9 ± 11%) and other non-sulfur microorganisms (Supplementary Dataset 1–4). Overall, average Shannon Diversity Indices (H’) decreased from 2.5 in 2015 to 1.9 for 2016/2017 and increased to 3 in 2018 (Supplementary Dataset 1).

The pH depth profiles showed circumneutral conditions throughout the water column in July 2015 and July 2018 (ranging from ~8.6 to 6.2), while July 2016 and July 2017 impoundment waters were more acidic (ranging from ~6.6 to 4.7) (Fig. 1d; Supplementary Dataset 2). The lowest pH values were observed in upper waters during the summer months of 2016 and 2017 (Fig. 1d). Spring dissolved oxygen concentrations in 2016 (May concentrations ranging from 0.11 to 0.21 mM; data for May 2017 not available) were considerably higher than those observed in spring 2015 and 2018 (May concentrations ranging from 0.006 to 0.09 mM) (Fig. 1e; Supplementary Dataset 2). July oxygen profiles across all years showed the establishment of steep oxygen gradients in the summer thermally stratified water cap. The epilimnetic upper water (~0–2 m) oxygen concentrations ranged from 0.17 to 0.24 mM and the lower metalimnetic and hypolimnetic waters (>3 m depth to 38 m) oxygen concentrations ranged from 0.03 mM to <limit of detection (LOD) (Fig. 1e; Supplementary Dataset 2).

Total sulfur (TotS) increased over the four years of investigation in the summer waters (Supplementary Dataset 3). [SO_4_^2−^] and [reactive S] (S_react_ = TotS − SO_4_^2−^, i.e., all possible S atoms that can take part in oxidative or disproportionation reactions^46^) were variable over time showing no evident temporal trends. The mean thiosulfate concentrations were 0.2 ± 0.3 mM. Dissolved aqueous sulfide (∑H_2_S) concentrations ranged from <1 μM to 6 μM progressively increasing with depth. The acidity to sulfate ratio ([H^+^]/[SO_4_^2−^]) provides a comparative indicator of whether microbial sulfur oxidation metabolism occurs via straight path oxidation or by disproportionation^47^. Higher values indicate greater relative oxidation of reduced sulfur compounds to sulfate accompanied by higher acidity generation, whereas lower values are indicative of more complex sulfur recycling (and often acid consuming) reactions. Comparison of [H^+^]/[SO_4_^2−^] values revealed significantly higher values in 2016 compared to any of the other three years (ANOVA and a post-hoc Tukey comparison test; *p* < 0.05) (Fig. 1f). [H^+^]/[SO_4_^2−^] values were also elevated in 2017, which is consistent with the lower pH values observed in 2016/2017. Thiosulfate concentrations in 2018 were statistically higher (*p* < 0.01) than those measured in 2016 and 2017 but were not statistically significantly different from 2015 thiosulfate concentrations (ANOVA and a post-hoc Tukey comparison test; *p* < 0.01) (Fig. 1g).

These results indicate clear trends of pH, concentrations of oxygen and a key S substrate, thiosulfate, associated with SOB community changes occurring in this system. *Halothiobacillus* was the dominant SOB in 2016 and 2017, which was associated with lower overall diversity (Supplementary Dataset 1 and 4), acidification (Fig. 1d, f), higher spring water cap oxygen concentrations (Fig. 1e) and lower thiosulfate concentrations (Fig. 1g). In contrast, significantly higher proportions of *Sulfuricurvum*, *Thiobacillus* and *Sediminibacterium* were detected in 2015 and 2018 water samples, associated with lower abundance of *Halothiobacillus* sp. (Fig. 1a), higher overall diversity (Supplementary Dataset 1 and 4), lower acidity (Fig. 1d, f) and spring oxygen concentration (Fig. 1e) and higher thiosulfate concentrations (Fig. 1g).

### Microbial community composition and functional genes

The results above suggest that the SOB communities present in this tailing impoundment catalyze sulfur oxidation through different pathways, resulting in different water chemistry outcomes. The depiction of sulfur pathways throughout this study were based on Watanabe et al.^28^ which in turn is based on the current collective understanding of microbial sulfur oxidative pathways. The aim was not to show all intricacies of each individual pathway set and various combinations but rather utilize this over-simplified but well informed overarching framework to aid in the determination of environmental occurrence and controls of these core known pathways. Based on sulfur metabolizing genes present in SOB genera, Sox and S4I sulfur oxidation pathways were possible across all years while the rDSR pathway was only possible in 2015 and 2018. (Fig. 2a). Most notably *Halothiobacillus, Sulfuricurvum, Thiomonas* and *Sulfurovum* possessed genes encoding the complete Sox pathway, *Thiobacillus* contained the genes for both the incomplete Sox and rDSR pathways and *Halothiobacillus, Thiobacillus, Sediminibacterium, Thiomonas, Brevundimonas* and *Caulobacter* possess the TsdA gene that catalyzes reactions known to occur in the S4I pathway (Fig. 2a). At the community level over the four years of the study, *soxXYZABCD* complex genes were consistently present while *dsrABCEFH, sat and aprAB* were limited to 2015 and 2018 (Fig. 2b; Supplementary Dataset 5) when *Thiobacillus* groups were present. TsdA was also consistently present across time and space, while *ttrABC* showed a similar trend to the *rDSR* gene that was only present in 2015 and 2018 (Fig. 2b).

A direct comparison of gene distribution and *Halothiobacillus* and *Thiobacillus* abundance, with [S_2_O_3_^2−^] (*n* = 30) and [H^+^]\[SO_4_^2−^] values revealed relationships between the abundance of these two major SOB genera in this system with overall sulfur geochemical outcomes and sulfur gene distributions (Fig. 2b–e). The 2015 and 2018 waters hosted microbial communities with functional S genes encoding the Sox (SoxXYZABCD), S4I (TsdA and TetH) and rDSR (DsrABCEFH), AprAB and Sat and had lower acidity to sulfate ratios and higher thiosulfate concentrations. Geochemical monitoring of the input waters discharging into the tailing impoundment over the course of this study revealed no discernible change in overall acidity loading over the course of this study^16^. These results imply greater overall sulfur recycling/disproportionating and/or slower oxidative reactions occurring in 2015 and 2018 relative to 2016 and 2017. In 2016 and 2017 the highest [H^+^]\[SO_4_^2−^] ratios and lowest [S_2_O_3_^2−^] (Fig. 2d, e) were coincident with the most abundant S genes encoded by the Sox (SoxXYZABCD) and S4I (TsdA catalyzed reaction) pathways (Fig. 2b). The geochemical trends of this comparison were consistent with patterns observed from the larger geochemical dataset ([H^+^] \ [SO_4_^2−^], *n* = 51; and [S_2_O_3_^2−^], *n* = 50) (Fig. 1f, g). In 2015 and 2018, when *Sulfuricurvum*, *Thiobacillus* and *Sediminibacterium* were dominant, the abundances of genes encoding the rDSR pathway were inversely correlated with oxygen through depth (Supplementary Fig. S6).

**Fig. 3:**
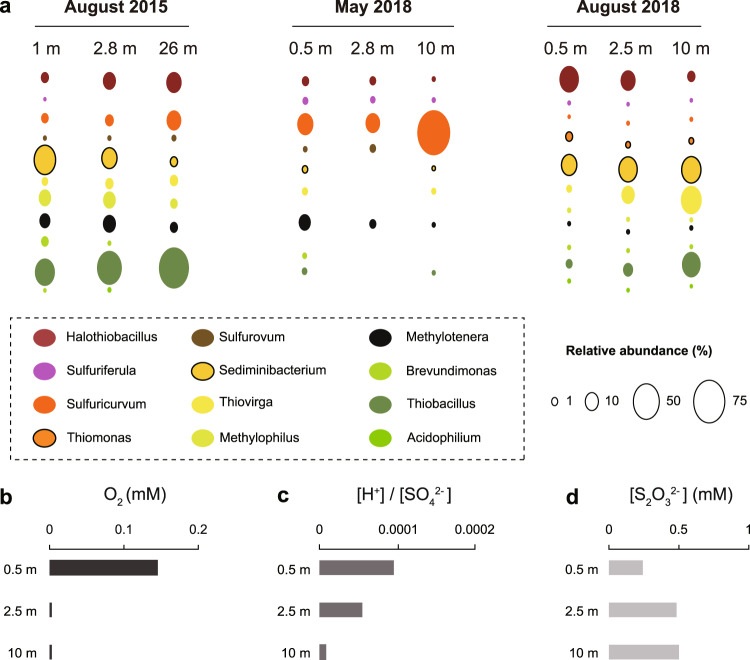
Oxic, hypoxic and anoxic zonations of SOB communities, their respective gene based sulfur oxidative and TEA reduction strategies and aqueous geochemical outcomes. **a** SOB relative abundances in August 2015, May 2018 and August 2018 through depth. **b** August 2018 dissolved oxygen concentrations (mM) through depth. **c** August 2018 H^+^/SO_4_^2−^ ratios throughout depths. **d** August 2018 thiosulfate concentration (mM) through depth. SOB, sulfur oxidizing bacteria.

SOB enriched directly from in situ mine waters provide an opportunity to examine gene driven differences in sulfur oxidation rates and sulfur intermediate formation under laboratory settings that can inform field study results (Fig. 3). Here, metagenomic and geochemical analysis of SOB enrichments grown from waters collected at this site and three other mine tailings impoundments revealed the same connections between genera, Sox vs. rDSR gene presence and sulfur outcomes as identified in our field results (Supplementary Fig. S3a, b). SOB enrichments that solely contained Sox genes had faster oxidation rates and did not produce detectable sulfite compared to SOB enrichments where rDSR genes were also present (Supplementary Fig. S3c, d). SoxAX genes were consistently the most abundant in all enrichments, which may be due to SOB possessing multiple copies of these genes as has been observed in a previous study^48^.

### Genomic and metabolic divergences of impoundment bacteria

**Fig. 4:**
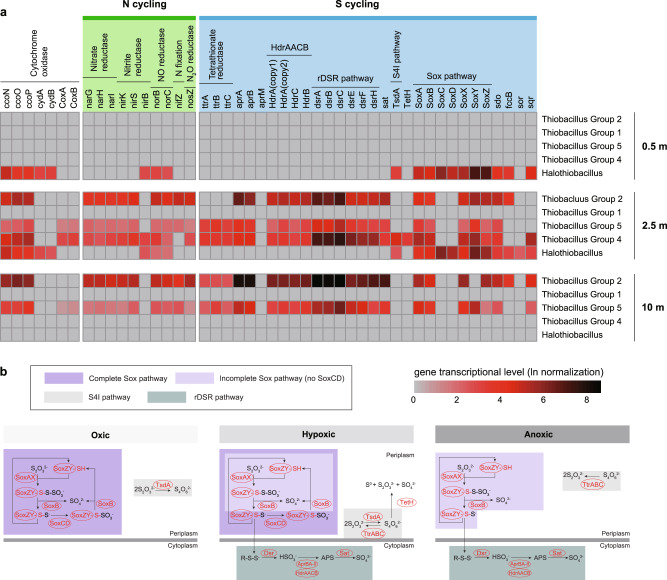
RNA expression activities and sulfur oxidation strategies revealed by metatranscriptomics. **a** August 2018 RNA expression levels of sulfur oxidative and oxygen/nitrogen compound reductase genes of *Halothiobacillus* and *Thiobacillus* groups through depth. **b** Oxic, hypoxic and anoxic zonations of dominant sulfur oxidative pathways based on the RNA expression levels.

Genome-resolved metagenomic analysis was used to reconstruct genomes of bacteria from the four most dominant genera and other species with genes for sulfur-oxidizing pathways. The genomic and metabolic diversity of the dominant sulfur cycling bacteria were compared. *Halothiobacillus* comprising a single group had very similar genomes (24 genomes sharing >99% nucleotide identity) that encode the Sox complex, Sor (sulfur oxygenase reductase), Sdo (sulfur dioxygenase), TsdA (thiosulfate dehydrogenase) and FccB (flavoprotein subunit of flavocytochrome) proteins of S metabolism (Fig. 2a and Supplementary Fig. S2). In contrast, far greater diversity existed within the *Thiobacillus* genus (17 genomes). Seven *Thiobacilli* groups were present in the tailing impoundment in 2015 and Groups 1, 3, 6, and 7 dominated, while in 2018 Groups 2, 4 and 5 dominated. All *Thiobacillus* genomes encoded Dsr (dissimilatory sulfur reductase), Apr (adenosine 5′-phosphosulfate reductase), Sat (sulfate adenylyltransferase), Sdo and an incomplete Sox (lacking SoxCD) complex (Fig. 2a), however cross group variation was evident in whether they possessed the *tsdA* gene (Fig. 2a and Supplementary Fig. S2). Both *Halothiobacillus* and *Thiobacillus* groups possessed the genomic capacity for aerobic respiration (CcoNOP) (Supplementary Fig. S2). Though two lineages of AprBA have been reported in *Thiobacillus* spp.^35^, here, the three groups (Groups 2, 4, and 5) of *Thiobacillus* spp. which dominated the 2018 samples only encoded the lineage II AprBA without AprM, while the other groups of *Thiobacillus* genomes (groups 1, 3, 6, and 7) encoded both lineages of AprBA (Fig. 4). Moreover, the TetH gene was only detected in group 4 *Thiobacillus* genomes.

*Sulfuricurvum* comprises two distinct groups (12 genomes), all with genomes that encode the Sox complex. *Sediminibacterium* formed four groups (13 genomes) and each genome contains the *sdo* and *tsdA* genes. Members of the *Sediminibacterium* genus have been reported as iron oxidizing bacteria^49–51^; however, here metagenomic analyses revealed they possess the *sdo* gene, suggesting they could be involved in sulfur oxidation as well. Other less abundant genera (*Brevundimonas*, *Methylotenera*, *Sulfurovum* and *Thiomonas*) possessed the capacity for sulfur oxidation through the Sox pathway (Fig. 2a and Supplementary Fig. S2).

Throughout time, there was a ubiquitous presence of metal tolerant (photo)-heterotrophs from the Sphingomonadaceae family (Fig. 1a). The photosynthetic abilities of Sphingomonadaceae members (*Sphingomonas*, *Novosphingobium* and *Sphingobium*) were confirmed with the presence of photosynthetic genes (Supplementary Dataset 5 indicates the elevations in these genes during 2016 and 2017) and their heterotrophic capabilities were inferred through the lack of autotrophic/carbon fixation genes, and extensive literature support these organisms are well known metal tolerant heterotrophs^52–54^.

### Transcriptional activities of metabolic genes

Since the presence of genes encoding S metabolic proteins alone does not ensure a change in sulfur geochemical dynamics as the pathway must be expressed, we isolated mRNA and conducted RNA-seq analysis of samples collected at three depths throughout water column in the peak summer stratified period of August 2018 (depth of 0.5 m, 2.5 m, and 10 m). Results showed that the patterns of abundance (Fig. 3a) and gene expression of *Halothiobacillus* sp. and *Thiobacillus* spp. (Groups 2, 4 and 5 were present at this time) (Fig. 4a) are consistent with the hypothesis that segregation of microbial sulfur oxidation metabolic pathways occurs across this steep oxygen gradient that results in different geochemical outcomes (Fig. 3b–d). The results of RNA-Seq analysis support the expression of three sulfur oxidation pathways: Sox, rDSR and S4I. *Halothiobacillus* activity, including Sox and S4I Sulfur pathways, and aerobic respiration genes (*ccoNOP*, *cydAB*), was mostly limited to the upper oxygenated epilimnetic waters (Fig. 4a). In particular, *Halothiobacillus* Sox gene expression was mostly limited to the oxic portions of the stratified water cap (epilimnetic and upper metalimnetic regions) (Fig. 4a), despite its detected occurrence within genomes of *Halothiobacillus* in anoxic hypolimnetic regions (Figs. 1a, 3a and Supplementary Fig. S2). In contrast, gene expression of *Thiobacillus* spp., including genes encoding the rDSR pathway, an incomplete Sox (lack of SoxCD) and S4I (TsdA) was confined to the low oxygen (2.5 m at 0.0016 mM O_2_) metalimnion (Groups 2, 4 and 5) and upper hypolimnion (10 m at 0.0006 mM O_2_) (Groups 2 and 5) regions (Fig. 4a). The detection and expression of TetH was only observed for Group 4 *Thiobacillus* from the 2.5 m depth sample. In line with this position in the water column, we observed the expression of genes encoding enzymes for nitrate reduction (*narGHI*), nitrite and nitric oxide reduction (*nirBK* and *norBC*) as well as sulfur oxidation (*DsrABCEFH*, *sat*, *aprAB*) in *Thiobacillus* spp. in the microoxic metalimnion and anoxic hypolimnion regions (Fig. 4).

The strongest patterns of expression of *DsrABCEFH*, *aprAB* and *sat* genes (possessed by *Thiobacillus* spp) (Fig. 2a) were in the micro-oxic and anoxic portions of the water column. Supporting these trends were the greater abundance of these functional genes within the micro-oxic to non-detectable oxygen (<LOD to 0.003 mM) portions of the water column in March 2015, August 2015 and August 2018 (Supplementary Fig. S6). This depth dependent segregation of SOB and thus sulfur pathways associated with decreasing oxygen concentrations are related to a clear differentiation of sulfur cycling outcomes associated with the shift from *Halothiobacillus* in the upper, more oxygenated waters to *Thiobacillus* in the metalimnetic, micro-oxic (Groups 2, 4 and 5) and deeper anoxic waters (Group 2 and 5). Specifically, decreasing acidity generation and increasing thiosulfate concentrations occurred with depth associated with these shifts in which SOB are present (Fig. 3a). While a lack of biological replicates for each depth sampled precludes statistical analyses, our experience^12,13^ and other numerous published studies^55–57^ have demonstrated the reliability of metatranscriptomic analyses used here for single replicate interpretation. Further, the differences observed in gene expression of our three spatially resolved RNA samples collected across the depth profile, are consistent with the other lines of evidence in the study (i.e., 16S rRNA, whole genome, geochemistry, physico-chemistry), in collectively demonstrating oxygen partitioning of SOX vs rDSR sulfur oxidation pathways.

## Discussion

**Fig. 5:**
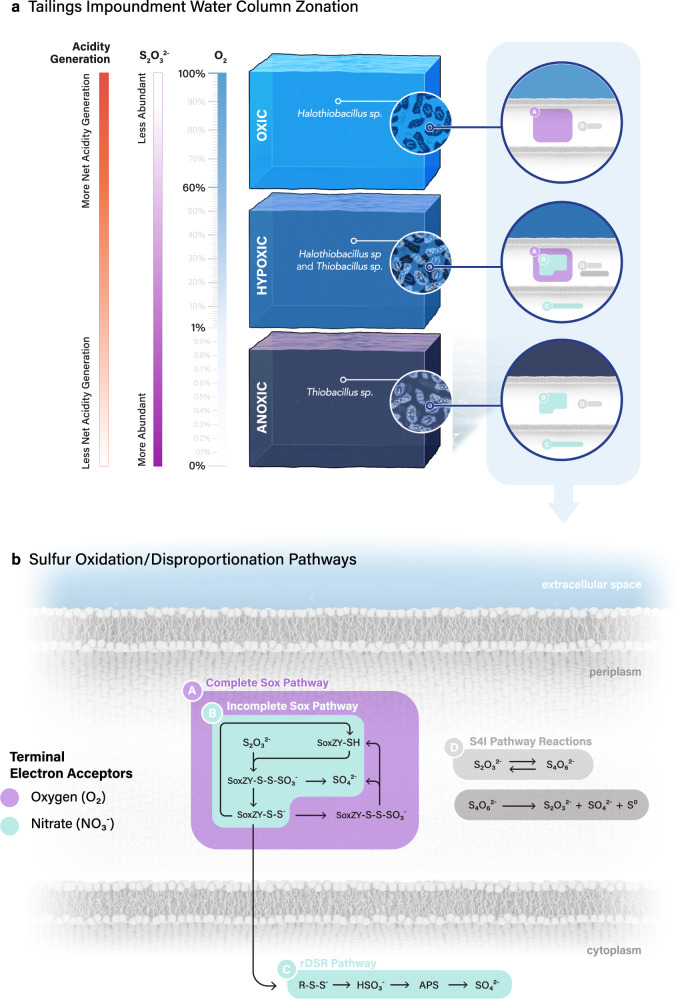
Conceptual model of differential microbial sulfur oxidative strategies throughout steep oxygen zonations. **a** Dissolved O_2_ zones, and dominant microbial oxidative strategy, based on gene expression, in the oxic zone (Sox driven oxidation coupled to O_2_), hypoxic zone (Sox-driven oxidation coupled to O_2_ and rDSR-driven oxidation coupled to NO_3_^−^) and anoxic zone (rDSR-driven oxidation coupled to NO_3_^−^) along with the different expected environmental water geochemical outcomes (acidity generation and thiosulfate abundance). **b** Sulfur oxidative strategy biochemical pathways and cellular location. Scientific illustration and visualization by Mark Belan (www.artscistudios.com).

The results of this study identify the importance of neutrophilic chemolithoautotrophic SOB in controlling sulfur cycling and associated acidification outcomes within tailing impoundment waters. The tailing impoundment water was consistently inhabited by neutrophilic chemolithoautotrophic SOB and (photo)-heterotrophs (Figs. 1a and 5). The sulfur oxidizing portion of the communities included members of the genera *Halothiobacillus*, *Sulfuricurvum, Thiobacillus, Thiovirga, Sulfurovum* and *Brevundimonas* that have previously been implicated in sulfur oxidation^16,58–64^. *Halothiobacillus* and *Thiobacillus* species from this study have no known representatives in current databases indicating they are novel members of these genera. Notably, the microbial community inhabiting the tailing impoundment waters (pH ~7 to 4) shared very few similarities with mining associated AMD communities present under more acidic conditions^2,65^. This result is not surprising given that pH has a strong effect on microbial community structure^21^. None of the typical ferrous and sulfur oxidizing species found to dominate in acidic mining waters (e.g., *Acidithiobacillus thiooxidans*, *Acidithiobacillus ferrooxidans*^2^ occurred in these circumneutral high sulfur waters (Fig. 1a). In fact, the communities analyzed in this study possessed similar genera found in systems experiencing accelerated metal/concrete corrosion^66^. Further, Lopes et al.^17^ identified both *Halothiobacillus* and *Thiobacillus* as dominant genera present in circumneutral tailings slurries at an active mine site in Portugal, suggesting that these sulfur oxidizers are highly adapted to these high metal and sulfur environments globally. With the exception of certain members of *Thiobacillus*, such as *Thiobacillus ferrooxidans*, whose presence and activity has been noted in AMD contexts^67^, the dominant neutrophilic SOB identified here, i.e., *Halothiobacillus, Sulfuricurvum*, *Sediminibacterium* and other *Thiobacillus* spp., were largely underappreciated as important organisms mediating oxygen consumption, sulfur compound transformation and acidity generation in mining impacted waters, prior to this study. In particular, the relationships between the distributions of these SOB, their metabolic functional genes and geochemical outcomes in environmental settings have not been well delineated. The relative proportions of various SOB and community level occurrence of sulfur metabolic genes were associated strongly with different sulfur geochemical outcomes, particularly with generation of acidity and the concentration of thiosulfate, a ubiquitous mine tailings associated S compound and important SOB S substrate.

This study supports the notion that higher oxygen conditions can drive the proliferation of *Halothiobacillus* and when present in high abundance, they drive progressive increases in net acid production. In 2016 and 2017, more oxygenated spring water column conditions may have facilitated the proliferation of *Halothiobacillus*, using a straight-path sulfur oxidation strategy through the Sox pathway, enabling *Halothiobacillus* to outcompete *Thiobacillus*, (incomplete Sox and rDSR pathways) (Fig. 1). Coincident to the years when *Halothiobacillus* was the dominant SOB (2016 and 2017), lower thiosulfate concentrations and significantly higher H^+^/SO_4_^2−^ ratios occurred in the water column (Figs. 1 and 2). *Halothiobacillus* has been proposed as an early indicator of net acid generating conditions in circumneutral waters^16,68^ as it has been found in higher abundance in moderately acidic (pH ~4) waters discharged from an active mine into the receiving environment and demonstrated to mechanistically drive these changes in laboratory incubations of mining waters^16^. The *Halothiobacillus* genus directly oxidizes reduced sulfur compounds to sulfate through the Sox pathway (Fig. 2) without formation of free sulfur oxidation intermediates^5,28^. However, as *Halothiobacillus* also encodes the *tsdA* gene (Fig. 2), this organism may also use the S4I pathway, and this is supported by expression of the *Halothiobacillus* t*sdA* gene in the epilimnetic (oxygenated) and metalimnetic (low O_2_) region of the water cap (Fig. 4). Consistent with the connection to spring conditions observed over time in this study (Fig. 1c), RNA-seq analysis in August 2018 confirmed that *Halothiobacillus* metabolic activity was limited to the epilimnetic and to a lesser degree, the upper metalimnetic portion of the water column, where higher oxygen concentrations were present (Fig. 4).

*Halothiobacillus*-dominated enrichments have been observed by the authors in previous work to rapidly drive circumneutral pH conditions to acidic levels under aerobic conditions^16,68^. Other studies have shown that acidity may be generated by the production of CO_2_ during the respiration of organic matter by heterotrophic communities^69^ and slightly lower organic carbon concentrations were observed in 2016 and 2017 (Supplementary Dataset 6), thus, some acidity generation may be attributed to aerobic heterotrophy. However, organic carbon concentrations are relatively low in this system compared to reduced sulfur (Supplementary Dataset 3) making acidity generation by microbial sulfur oxidation the more likely scenario. In addition, enrichment and genomic results collectively provide strong evidence that *Halothiobacillus* is capable of driving circumneutral conditions to lower pH through a direct sulfur oxidation pathway, resulting in sulfate and acid in mine water systems when oxygen is present. However, not all s*oxABCDXYZ* encoding microbes were shown to be capable of acidity generation akin to *Halothiobacillus*. *Sulfuricurvum* found in this system, like *Halothiobacillus*, contains genes for the complete Sox complex (s*oxABCDXYZ*) and no S4I, rDSR or HDR encoding genes (Fig. 2a), and its relative abundance in 2015 was similar to *Halothiobacillus* abundance in 2016/2017 (Fig. 1a). However, its presence in 2015 did not coincide with any significant acidity generation (Fig. 1).

The relationship between higher spring oxygen concentrations and the presence of *Halothiobacillus* does not explain its continued dominance as anoxic/micro-oxic conditions are established in the metalimnion and hypolimnion during summer stratification (Figs. 1 and 3). Emerging evidence has shown that obligate aerobe*s* under anoxic conditions may participate in syntrophic cryptic oxygen cycling utilizing a heterotrophic partner that can generate molecular oxygen^70,71^. However, an inverse correlation between *Halothiobacillus* and Sphingomonadaceae through depth in 2016 and 2017 (Supplementary Fig. S5) suggest these genera occupy different ecological niches, with the latter occupying upper waters with light availability where photosynthetic *Sphingomonas*, *Novosphingobium* and *Sphingobium* would be favored.

In 2015 and 2018, the genes *dsrEBFH, aprAB* and *sat* along with genes encoding the incomplete Sox complex (lacking SoxCD) (possessed by *Thiobacillus*) were progressively more abundant with depth and decreasing oxygen conditions (Supplementary Fig. S6). RNA expression in August 2018 confirmed the enhanced expression of these genes by *Thiobacillus* corresponding with declines in dissolved oxygen (Fig. 4; Supplementary Dataset 7). Phylogenetic analysis comparing DsrAB present in this ecosystem to organisms shown to utilize the rDSR pathway supported that these proteins are being utilized for oxidative reactions by *Thiobacillus* rather than for reduction (Supplementary Fig. S4). Therefore, our results are well aligned with recent work showing that environmentally relevant chemolithoautotrophic sulfur cycling bacteria can mediate a reversal of the sulfate reduction pathway catalyzed through the Dsr complex to disproportionate, or oxidize reduced sulfur species^72–74^. This capability has also been identified in phototrophic sulfur bacteria such as *Allochromatium vinosum* (*Chromatiaceae* family)^72,74^^,^. Metagenomic characterization of SOB from this tailings impoundment strongly supports that rDSR pathways appear less efficient in generating acidity and sulfate compared to the Sox pathway, and there is potential for free sulfite formation through rDSR (Fig. 4b, c). In situ analysis of sulfite concentrations in the tailing impoundment were not consistent with increased sulfite formation when rDSR pathways were more abundant (Supplementary Dataset 5), but sources of sulfite beyond the rDSR pathways (i.e., directly from mill effluents) can overprint sulfite formation and utilization from biogeochemical cycling, particularly at these relatively low concentrations (Supplementary Dataset 3). In addition, the presence of the rDSR pathway coincided with the presence of genes encoding TtrABC (Figs. 2a and 3b, c), which may reduce tetrathionate, regenerating thiosulfate, as well as the presence of an incomplete Sox complex (lack of SoxCD).

The rDSR pathway is proposed to be more energy efficient than the Sox pathways^33^ likely evolving in the early Archean era when the ability to oxidize sulfur compounds under anoxic conditions would have been necessary^27^. Their increased prevalence (Supplementary Fig. S6) and higher gene expression (Fig. 4) with depth are consistent with these pathways dominating in the micro/anoxic depths of these tailing impoundment waters and their reactions may be coupled to both aerobic respiration and nitrate/nitrous oxide reduction. *Thiobacillus* expressed nitrate reduction genes at increased depth in August 2018 (Fig. 4). However, ammonia concentrations did not increase with depth (Supplementary Dataset 6), likely reflecting multiple sources of ammonia, such as ammonium nitrate from rock blasting or a by-product of the cyanidation process during ore recovery in mill processing. These allochthonous N sources are contributed during on-going discharges of mine impacted waters and tailings to the system, and may obscure a possible signal from denitrification. Nevertheless, the RNA-seq results (Fig. 4) suggest that several populations of *Thiobacillus* (Supplementary Fig. S2), observed during 2015 and 2018 summer months are coupling sulfur oxidation to denitrification under lower oxygen concentrations, deeper in the impoundment water column and across time in this system. The reactions catalyzed by these different pathways (Fig. 4b) will determine the occurrence, distribution and transformation of intermediate sulfur species within the impoundment (Figs. 1 and 3). These results provide new evidence that biological sulfur oxidation pathways are important in determining when and where higher concentrations of reduced sulfur compounds such as thiosulfate will occur.

An adaptive capability of indigenous organisms to couple sulfur oxidation to nitrate or nitrous oxide reduction is likely an energetically favorable and viable metabolic strategy in tailing impoundment waters where sulfur oxidation has driven waters micro-oxic/anoxic when oxidized nitrogen species are present (Fig. 5). Experimentally, it is well established that *Thiobacillus denitrificans* can utilize nitrate as a terminal electron acceptor^75^. In other studies, *Desulfurivibrio alkaliphilus* has been shown to be capable of oxidizing reduced sulfur species coupled to nitrate reduction using rDSR under anoxic conditions^72^. Similarly, in the deep terrestrial subsurface, the coupling of nitrate reduction and S oxidation via rDSR mediation has also been found to occur^76,77^. Thus, while there was a consistent presence of *soxABCXZ* and *tsdA* (possessed by *Halothiobacillus* and *Sulfuricurvum*) throughout depth and across time in this study, it appears that *Thiobacillus* may gain a competitive advantage over *Halothiobacillus* under low oxygen to anoxic conditions, using the more energy efficient rDSR pathways coupled to denitrification or small amounts of oxygen when available. In the in situ environmental investigations, *Thiobacillus* was utilizing rDSR pathways coupled to denitrification which would not be expected to result in extensive acidity generation. However, the results of our SOB enrichment experiments suggest that even under oxygenated and nitrate limited laboratory conditions, rDSR consortia can carry out sulfur oxidation of thiosulfate utilizing oxygen but at significantly slower observed rates (estimated by thiosulfate depletion) than their *Halothiobacillus* (Sox pathway) counterparts (Supplementary Fig. S3).

Our results enable the development of a conceptual integrated geochemical and biological framework of microbial sulfur oxidation in response to oxygen conditions in mining impacted waters receiving high sulfur loads, identifying the important SOB genera and pathways involved as well as the geochemical outcomes (Fig. 5). Oxygen dependent niches for SOB with different sulfur metabolizing strategies, i.e., Sox pathway (e.g. *Halothiobacillus*) vs. incomplete Sox and rDSR pathways (e.g. *Thiobacillus*), occur associated with their capacity to couple oxidation to terminal electron acceptors like O_2_, versus NO_3_^−^/NO_2_^−^ (Fig. 5). These different strategies mechanistically explain environmentally important outcomes of acidity generation and occurrence of a key sulfur substrate, thiosulfate. Depth dependent oxygen gradients segregate *Halothiobacillus* activity to upper, more highly oxygenated waters and their proliferation when higher spring overturn oxygen concentrations occur resulting in the lowest summer pH values and highest average thiosulfate concentrations over the four years of the study (Figs. 1 and 5). Conversely, in the absence of detectable oxygen or under low oxygen conditions, i.e., at deeper depths in the water column, or during years when spring oxygen concentrations were lower (2015 and 2018), *Thiobacillus* groups were active coupling sulfur oxidation to O_2_ and/or nitrate, through the incomplete Sox and rDSR pathways resulting in higher pH values and thiosulfate concentrations (Fig. 5).

## Methods

### Site description

The tailings impoundment is the central repository for mining impacted water and sulfidic mineral tailings from active local mining operations with a total volume of 19,975,000 m^3^ (Supplementary Fig. S1a). This system was designed from a pre-existing lake to inhibit the oxidation of the deposited high sulfide tailings, yet encourage oxidation of aqueous sulfur oxyanions (e.g., S_2_O_3_^2−^, S_3_O_6_^2−^, S_4_O_6_^2−^) residing in the surface water layers of the impoundment to sulfate (the mill commonly inputs higher concentrations of SO_3_^−^ and S_2_O_3_^2−^) prior to discharge into a river system and local watershed^78^. This basin forms the beginning of a large-scale water treatment system at a Ni/Cu mine in Sudbury, Ontario, Canada^14,16^ with dynamic inputs from various sources including ore processing waters, tailings deposition and natural tributaries. The impoundment has a maximum depth of ~38 m and is dimictic, with summer (open water) and winter (under ice) thermal stratification periods and residence times estimated in the epilimnetic portions of the water column to be on the order of months.

### Water sampling and processing

Water samples were collected during 21 sampling campaigns from the impoundment at various depths over four years from March 2015 to August 2018. Samples occurred largely during open water periods (i.e., no ice cover, exception March 2015; Supplementary Dataset 1). The sampling procedures have been described in detail elsewhere^16,46^. Briefly, water samples were collected from specified depths during each campaign from a floating platform accessible by boat in ~38 m of water cap depth and if unavailable, from nearby shore locations. Prior to sampling, a physico-chemical profile of the water column was collected using a multiprobe (temperature, O_2_, pH, ORP, conductivity; YSI 600 XLM). Physicochemical survey data for all 21 sampling campaigns are provided in Supplementary Dataset 2. Water samples were collected using a VanDorn water sampling bottle (Wildco, 3.2 L Horizontal beta™) during stratified periods from the epilimnion, metalimnion and hypolimnion, and from upper and lower depths during mixing periods. In addition, during some campaigns surface water samples (0–0.5 m) were also collected from shore utilizing a telescopic sampler. Water samples were collected for geochemical analysis (*n* = 53; sulfur and nitrogen speciation, dissolved organic carbon (DOC)) and for molecular biology analysis (*n* = 30; 16S rRNA gene sequencing and metagenomics as well as metatranscriptomics (RNA) analysis in August 2018 (*n* = 3). A subset of these in situ waters were used in laboratory sulfur oxidative enrichment experiments along with wastewaters collected from three other mine sites in Canada (Flin Flon, Manitoba; Snow Lake, Manitoba; Baie Verte, Newfoundland). Detailed methods, geochemical and 16S rRNA gene sequencing results of enrichments were described previously^16^. Here, we present the metagenomic results of those SOB enrichments to align with the field-based metagenomes.

Water samples for SO_4_^2−^ and nitrogen species (NH_4_^+^, NO_2_^−^, NO_3_^−^) quantification were collected in 250 ml to 1 l polypropylene bottles (each rinsed three times with site water prior to collection) and completely filled to ensure no headspace and stored at 4 °C prior to analysis (within 24–72 h). ΣH_2_S was determined on-site rapidly after sample collection as previously described^46^. Thiosulfate (S_2_O_3_^2−^) and sulfite (SO_3_^2−^) analytes were stabilized immediately upon sampling through derivatization with monobromobimane^79^. In detail, water samples (100 μL) were directly pipetted into 2 mL glass amber vials with derivatization agents (100 μL of acetonitrile, 100 μL of 50 mmol L^−1^ HEPES (4-(2-hydroxyethyl) piperazine-1-ethanesulfonic acid, ≥99.5%, Sigma), 5 mmol L^−1^ of EDTA buffer (ethylenediaminetetraacetic acid, 99.4–100.6%, Sigma Aldrich; pH = 8.0, adjusted with NaOH) and 20 μL of 48 mmol/L monobromobimane (>97%, Sigma Aldrich). After 30 min of reaction time, 200 μL of methanesulfonic acid (~100 mmol) was added (≥99.5%, Sigma Aldrich) and samples were subsequently kept frozen until analysis. For total sulfur (TotS) analyses, water samples were collected in triplicate (40 mL) in situ and filtered through Pall Acrodisc® 25 mm 0.45 μm Supor® membrane filters with polypropylene syringe into 50 Falcon^TM^ tubes pre-spiked with acid to achieve final concentrations of 0.2% HNO_3_ (Optima grade, Fisher Chemical) and then stored at 4 °C until further processing. Water samples for TOC and DOC concentrations were collected in acid washed amber glass bottles pre-combusted at 450 °C for 8 h and frozen until analysis. Samples for DNA and RNA analyses were filtered (~2 to 5 L) through triplicate 0.2 and 0.1 μm filter towers (Thermo Scientific™ Nalgene™ Rapid-Flow™ Sterile Disposable Filter Units with CN Membrane) until filters clogged. Filters were excised and kept frozen (−20 °C for DNA, −80 °C for RNA) until nucleic acid extraction.

### Genomic DNA extraction and quantification

The DNA extraction was conducted on filters using DNeasy PowerWater DNA Isolation Kit (QIAGEN), and isolation was carried out with recommended protocols (QIAGEN). DNA extracts were sent to the Farncombe Metagenomics Facility at McMaster University, (Hamilton, Ontario) for subsequent analysis. Library concentrations were quantified through quantitative PCR (qPCR) and concentrations were adjusted as appropriate at each step of the molecular protocol.

### RNA extraction, library generation and sequencing

Samples for RNA analyses were collected at three depths (0.5 m, 2 m, 10 m) on August 14, 2018. These spatial RNA samples were collected via filtering the water as described above. For each sample, a total of ~2 to 5 L of water were filtered depending on when the filter clogged (total filtering time <60 min) at ambient temperature and were immediately (within 30 s) preserved at −80 °C before RNA extraction. The extraction of total RNA was conducted on filters using the RNeasy PowerWater Kit (from Qiagen). The concentration of total RNA was determined using a Nanodrop instrument and the quality of the preparation was assessed by agarose gel electrophoresis to monitor 16S and 23S ribosomal RNA. Samples were stored at −80°C; samples from two extractions were pooled before library construction. Quality controls, rRNA depletion, cDNA library construction from isolated RNA and sequencing were performed at the Génome Québec Innovation Centre (Montréal, Canada). Sequencing of the libraries was done using Illumina NovaSeq technology (NovaSeq 6000 S4, 100 bases paired-end).

### Barcoded 16S rRNA gene amplicon sequencing and bioinformatics analyses

Aliquots of purified DNA were used to amplify region V4 of the 16S rRNA gene by PCR using Illumina adapted primers following Bartram et al.^80^ and standard protocols of the Earth Microbiome Project^81,82^. In short, the primers were modified to amplify 515f (GTGYCAGCMGCCGCGGTAA) and 806r (GGACTACNVGGGTWTCTAAT) variable regions of archaeal and bacterial 16S rRNA gene. PCR was performed using 50 ng of the template and the PCR mix containing 1U of recombinant Taq DNA Polymerase (Invitrogen™), 1× buffer, 1.5 mmol L^−1^ MgCl2, 0.4 mg mL^−1^ BSA, 0.2 mmol L^−1^ dNTPs, and 5 pM of each primer. The reaction was carried out at 98 °C for 5 min, 35 cycles (98 °C) for 30 s, then 30 s (50 °C) and 30 s (72 °C), with a final extension of 72 °C for 10 min. PCR products were checked by electrophoresis. All amplicons were normalized using the SequalPrep normalization kit (ThermoFisher#A1051001) and sequenced using the Illumina MiSeq platform. Raw sequences were filtered and trimmed with a minimum quality score of 30 and a minimum read length of 100 bp using Cutadapt^42,83^. DNA sequence reads were filtered and trimmed based on the quality of the reads for each Illumina run separately, error rates were learned and sequence variants were determined by DADA2 version 1.6.0^84^. SILVA database version 132^85^ was used to remove bimeras and assign a taxonomy based on 16S rRNA sequences.

### Metagenomic sequencing, reads processing and assembly

The genomic DNA samples extracted from filters (duplicates of those used for 16S rRNA gene sequencing analyses) as well as preserved DNA extracts (*n* = 9) from sulfur oxidation enrichments described in Whaley-Martin et al.^16^ were used for metagenomic sequencing. The DNA extracts were dried and resuspended in 25 μL of water. Construction of libraries (insert length ~500 bp) and sequencing by Illumina HiSeq 1500 with paired-end 150 bp sequencing kit, were performed at the Farncombe Metagenomics Facility at McMaster University. The raw paired-end reads of the metagenomic sequencing samples were filtered to remove Illumina adapters, PhiX and other Illumina trace contaminants with BBTools^86^, and low quality bases and reads using Sickle (version 1.33). The de novo assembly was performed with IDBA_UD^87^ (parameters:–mink = 20,–maxk 140,–step 20,–pre_correction). In order to determine the sequencing coverage of each scaffold from a given sample, quality reads sets from all samples were individually mapped to the full assembly using Bowtie2^88^ with default parameters. The sam file was converted to bam format and sorted using samtools^89^, and the coverage of each scaffold from a given sample across all samples was calculated using the jgi_summarize_bam_contig_depths script from MetaBAT^90^. All the scaffolds with a minimum length of 2500 bp were assigned to genome bins using MetaBAT^90^, with both the tetranucleotide frequency and sequencing coverage across all samples considered. All the binned and unbinned scaffolds with a minimum length of 1000 bp were uploaded to ggKbase (ggkbase.berkeley.edu) for manual genome curation, based on GC content, sequencing coverage and taxonomic information of each scaffold, the basic idea of this process has been previously evaluated^91^, which was primarily based on the distribution and pattern of GC content and sequencing coverage of contigs in each bin.

### Gene prediction and annotation

The assembled scaffolds with a minimum length of 1 kbp (hereafter “1k_scaffolds”) were included for gene prediction and subsequent analyses. The protein-coding genes were predicted by Prodigal V2.6.3^92^ from 1k_scaffolds (parameters: -m -p meta). The 16S rRNA genes were predicted from 1k_scaffolds based on an HMM database reported previously^93^. The tRNAs encoded on all 1k_scaffolds were predicted using tRNAscanSE (version 2.0.3)^94^. The predicted protein-coding genes were compared against the databases of Kyoto Encyclopedia of Genes and Genomes (KEGG)^95^, UniRef100^96^ and UniProt^97^ using Usearch (version v10.0.240_i86linux64)^98^ for annotation. For specific metabolic potentials that are of interest, the predicted protein-coding genes were also searched against the HMM databases reported previously^99^, including the genes for key enzymes for respiration, photosynthesis, carbon fixation, nitrogen metabolism, hydrogen metabolism and sulfur metabolism. A recently published tool named DiSCo^100^ was used to distinguish the DsrC gene and its homologs. The sHdr genes were obtained by searching the predicted proteins against the sHdr protein sequences encoded by *Acidithiobacillus caldus* SM-1, *Thioalkalivibrio* sp. K90mix and *Hyphomicrobium denitrificans*^32^, using blastp and the results were manually checked to confirm. The HdrAACB genes were screened by checking the annotations from KEGG, UniRef100 and UniProt using the key word of “Hdr”, and the obtained list of genes were manually confirmed. The AprM genes encoded by *Thiobacillus* genomes in this study were identified by comparing all the protein-coding genes from the corresponding metagenomes against the AprM in *Thiobacillus denitrificans* (WP_011312796.1) using BLASTp with a e-value threshold of 1e−10, and hits manually confirmed. The TetH gene was identified by a BLASTp search as previously described^28^ followed by manual confirmation. The TetH gene was only detected in the *Thiobacillus* group 4 genomes, not in any of the *Halothiobacillus* genomes.

### Genome comparison

To identify the similarity of the genomes for each genus that is of interest, the “compare” function of dRep^101^ was used on the genomes modified by ggKbase (ggkbase.berkeley.edu).

### RNA-Seq analysis

The raw RNA sequencing reads were conducted for quality control as performed for metagenomic reads (see above). The quality RNA reads were mapped to the nucleotide sequences of protein-coding genes from the corresponding samples, using Bowtie2^102^ with default parameters. The transcriptional level of each protein-coding gene was determined by calculating its RNA sequencing coverage using the jgi_summarize_bam_contig_depths script from MetaBAT^90^.

### Relative abundance calculation of functional genes

To calculate the relative abundance of each protein-coding gene of interest, the total coverage of all genes in each sample was first determined as: Total_cov_ = *G*_*ij*_ x C_*j*_, where *G*_*ij*_ is the number (i.e., *i*) of protein-coding genes on scaffold *j*, and C_*j*_ is the sequencing coverage of scaffold *j* (see above). The coverage of a specific gene (for example, gene A) is determined as: Gene_A_cov_ = C_*j*_, where C_*j*_ is the sequencing coverage of scaffold *j* that encodes gene A. Thus, the relative abundance of gene A in the sample is determined as below:

$$Rel\_abu{n}_{A}={C}_{j}\, \times \,100/Tota{l}_{cov}\%$$

### Phylogenetic analyses

To distinguish the function (oxidation or reduction) of the detected *dsrAB* genes, a phylogenetic tree was built for concatenated DsrA and DsrB protein sequences, with references from Anantharaman et al.^34^. The DsrA and DsrB sequence sets were respectively aligned with MUSCLE^103^ and filtered using trimAl^104^ to remove those columns with at least 90% gaps, then concatenated using Geneious Prime^105^ in the order of DsrA-DsrB. The phylogenetic tree was built using IQtree^106^ with 1000 bootstraps and the “LG + G4” model. The oxidative type of DsrAB was determined according to Anantharaman et al.^34^.

### Sulfur and nitrogen speciation analyses

Dissolved ΣH_2_S, SO_4_^2−^, NH_4_^+^, NO_2_^−^, NO_3_^−^ concentrations were determined on unfiltered samples by colorimetry using a HACH DR1900 Spectrometer following the procedures recommended by the manufacturer (HACH Company, Loveland, CO, USA)^16,46,107^. Thiosulfate and sulfite concentration analyses (field stabilized monobromobimane derivatives) were carried out with methods adapted from ref. ^79^ on a Shimadzu LC-20AD prominence liquid chromatography (LC) system coupled to a fluorescence UV/VIS detector on an Alltima^TM^ HP C18 reversed phase column (150 mm × 4.6 mm × 5 μm, Grace^TM^) held at 35 °C using an isocratic mobile phase of 65% of 0.25% acetic acid v/v (pH 3.5 adjusted with NaOH) and 35% methanol at 0.5 mL min^−1^ for a total run time of 12 min as described previously^16^. The excitation wavelength was set to 380 nm and the emission wavelength 478 nm. Sulfite eluted at ~5.1 min and thiosulfate eluted at ~5.5 min. Calibration curves were prepared using sodium sulfite (Sigma Aldrich, ≥98% purity) and sodium thiosulfate (Sigma Aldrich, 99% purity). All sulfur compound concentrations reported represent the sulfur molarity (i.e. S in S_2_O_3_^2-^).

### Total sulfur analysis

Total sulfur (TotS) concentration analyses were conducted at the Commonwealth Scientific and Industrial Research Organization (CSIRO), Sydney, Australia by inductively coupled plasma optical emission spectroscopy (Varian730 ES, Mulgrave, Australia). Varian Fast Automated Curve-fitting Technique (FACT) was used to correct for background/inter-element interferences. Calibration standards in 2% v/v HNO_3_ were prepared from certified reference stocks (AccuStandard New Haven, CT, USA). The limit of detection (LOD) for TotS was 1 mg/L.

### Dissolved organic carbon analysis

Frozen samples were thawed and for dissolved organic carbon (DOC) analysis a sub-sample was filtered through Pall Acrodisc® 25 mm 0.45 μm Supor® membrane filters using polypropylene syringes and placed into carbon clean 40 mL glass vials. Total and inorganic carbon concentrations were determined on a Shimadzu TOC-L using a 680 °C combustion and non-dispersive infrared (NDIR) detection. Inorganic carbon samples were acidified (25% H_3_PO_4_) and sparged prior to combustion analysis using the inorganic carbon (IC) reactor kit attachment. Calibration standards were prepared using potassium hydrogen phthalate (Sigma Aldrich, 99.95–100.05% purity) for total carbon, sodium hydrogen carbonate (Sigma Aldrich, ≥99.7% purity) and sodium carbonate (Sigma Aldrich, ≥99.5% purity) for IC standards. DOC values were determined using subtraction of IC values from total carbon concentrations for each filter fraction (limits of detection for DOC were 1 mg/L C).

### Statistical and data analysis

Non Metric Dimensional Scaling (NMDS), Bray-Curtis Dissimilarity Clustering and Pearson R correlation matrices were carried out in R version 1.1.463 utilizing the Vegan package version 2.4-5. Chemical and biological data where concentrations/abundances were less than the limits of detection were treated as zero for statistical analysis.

## Supplementary information

Supplementary Information

Description of Additional Supplementary Files

Supplementary Dataset 1-7

## Data Availability

The 16S rRNA sequencing data, metagenome and metatranscriptome, and the metagenome-assembled genomes (MAGs) data generated in this study have been deposited in the NCBI database under accession code *PRJNA882497* via ID 882497 - BioProject - NCBI (nih.gov). All the MAGs are also available at ggkbase.berkeley.edu via https://ggkbase.berkeley.edu/mine_tailing_impoundment_time_series.
