# Supplementary material for: O2 partitioning of sulfur oxidizing bacteria drives acidity and thiosulfate distributions in mining waters
Source: Nat Commun. 2023 Apr 10;14:2006. doi: 10.1038/s41467-023-37426-8 (PMC10086054; doi:10.1038/s41467-023-37426-8)
Supplement: Supplementary file 1 — Supplementary Information [file 41467_2023_37426_MOESM1_ESM.pdf]

O_2 Partitioning of Sulfur Oxidizing Bacteria Drives Acidity and Thiosulfate Distributions in Mining Waters

Kelly J. Whaley-Martin, Lin-Xing Chen, Tara Colenbrander Nelson, Jennifer Gordon, Rose Kantor, Lauren E. Twible, Stephanie Marshall, Sam McGarry, Laura Rossi, Benoit Bessette, Christian Baron, Simon Apte, Jillian F. Banfield, Lesley A. Warren

Supplementary Figures

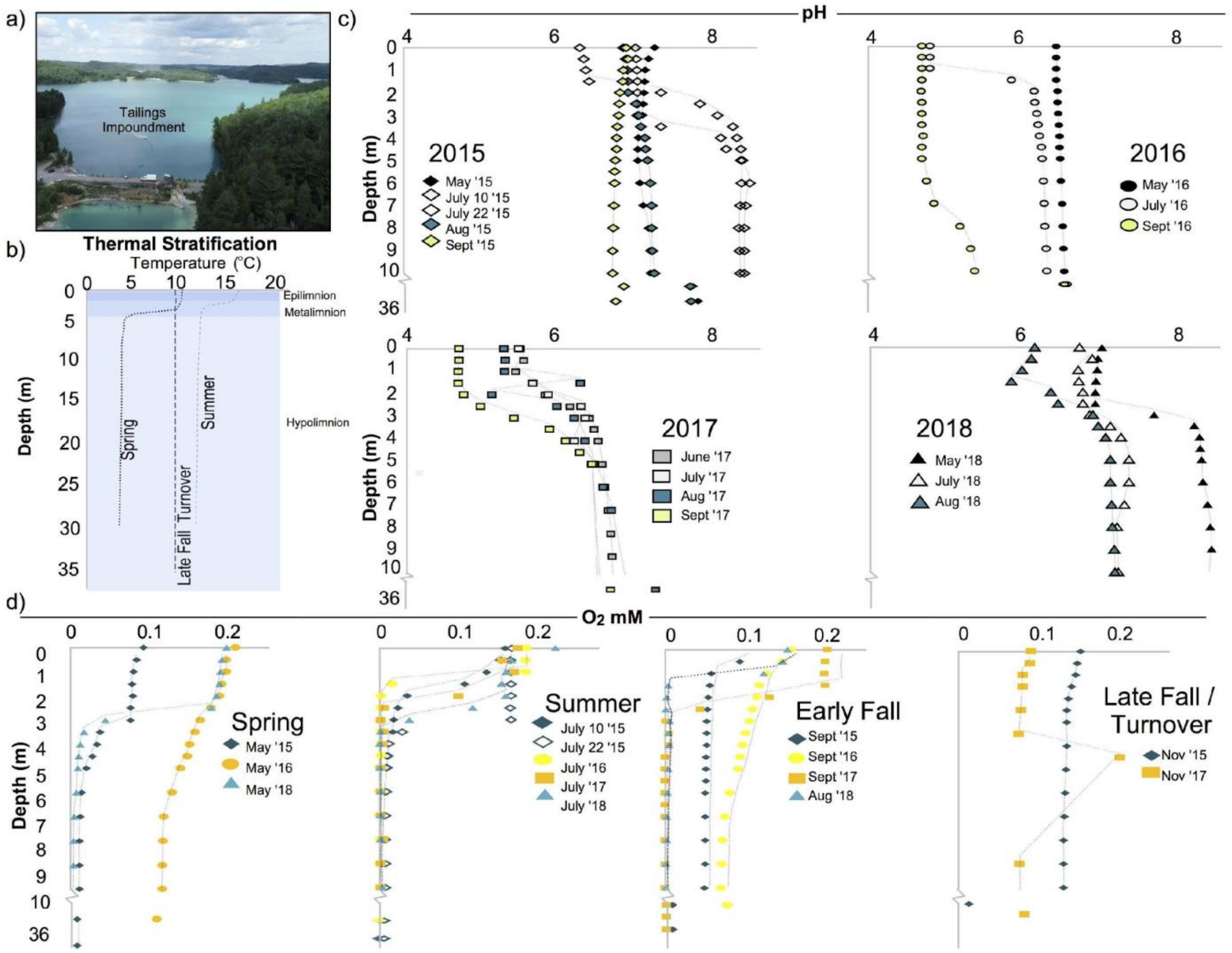

Fig. S1| Physiochemical depth profiles in tailing impoundment waters from 2015 to 2018 (0-10 m; comprising the epilimnion, metalimnion and upper hypolimnion when stratified, of the ~38 m water column). (a) Drone photograph of tailings impoundment b) Representative seasonal temperature profiles (Spring, Summer, Late Fall Turnover) for the dimictic tailings impoundment (c) Monthly pH depth profiles by year: 2015, 2016, 2017 and 2018. (d) Oxygen (mM) depth profiles by season across years: Spring (May), Summer (July), Early Fall (August, September) and Late Fall turnover (November).

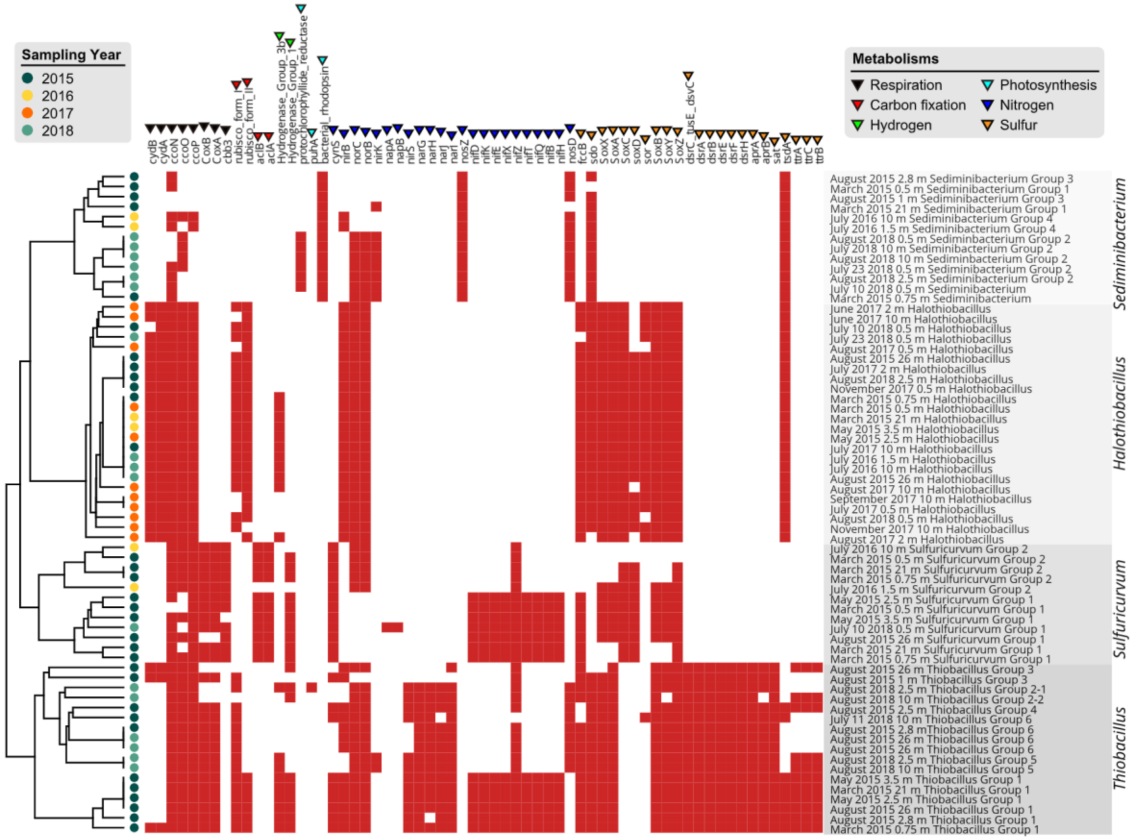

Fig. S2 | The distribution of genes in key metabolisms. The presence of a given gene in a genome is indicated by a red square, its absence by a white square. The genes are shown for the dominant sulfur oxidizing genera in the tailings impoundment waters, *Thiobacillus*, *Sulfuricurvum*, *Halothiobacillus* and *Sediminibacterium.* The functional categories of the genes are indicated by colored triangles. The genomes are clustered based on the presence and absence of listed genes.

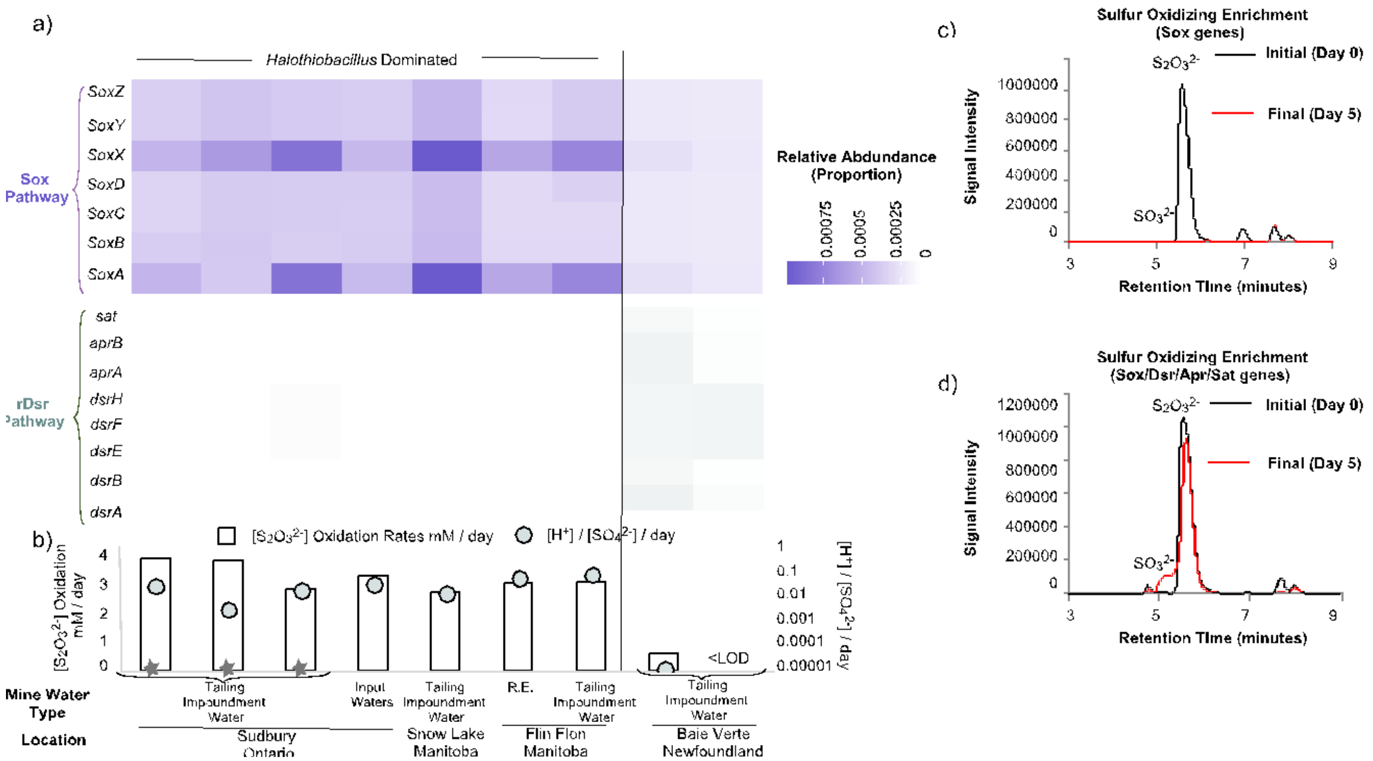

Fig. S3 |(a) community level gene relative abundances of sulfur oxidizing bacterial laboratory enrichment from four mine sites (*SoxABCDXYZ, dsrABEFH, aprAB* and *sat),* (b) daily rate of $S_2O_3^{2-}$ oxidation and rate change of $[H^+]:[SO_4^{2-}]$ / day, (c) and (d) the initial (day 0) and final (day 5) chromatograms (HPLC-UV/Vis) showing dissolved sulfite and thiosulfate in the sulfur oxidizing bacteria enrichment waters with (c) an enrichment with functional genes *soxABCDXYZ* and (d) an enrichment with *soxABCDXYZ* and *dsrABEFH*, *aprAB*, *sat.* genes (R.E. stands for receiving environment (water))

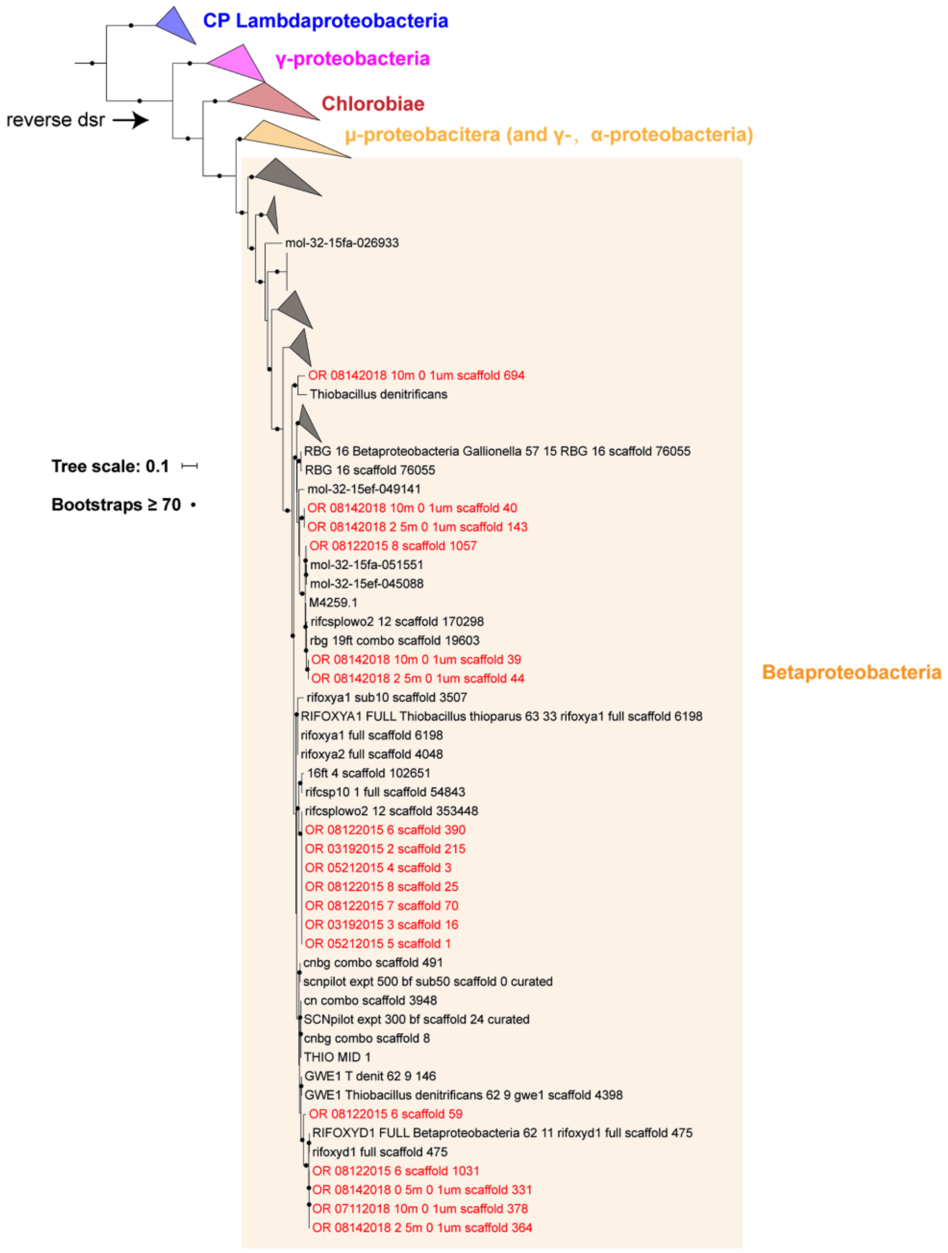

Fig. S4 | Concatenated DsrAB phylogenetic tree indicated the DsrAB identified in this study are likely responsible for sulfur oxidation (i.e., reverse dsr). The DsrAB sequences reported in this study are in red. See Methods for details of tree construction. Reference sequences are retrieved from [57].

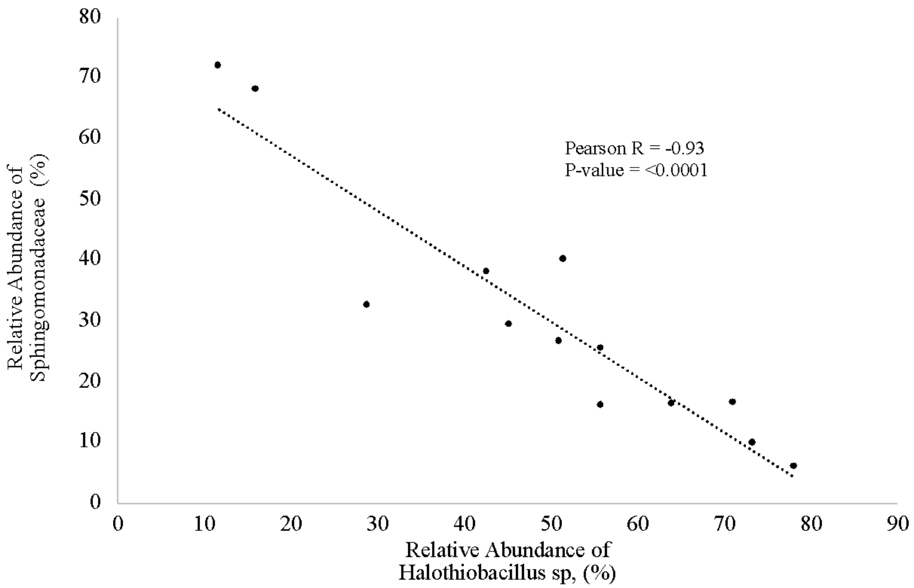

Fig. S5 | Relationship between the relative abundances of *Halothiobacillus* sp. and the *Sphingomonadaceae* family in 2016/2017 waters utilizing a two-tailed Pearson R correlational test (Pearson R = -0.93, p-value = 0.00000413).

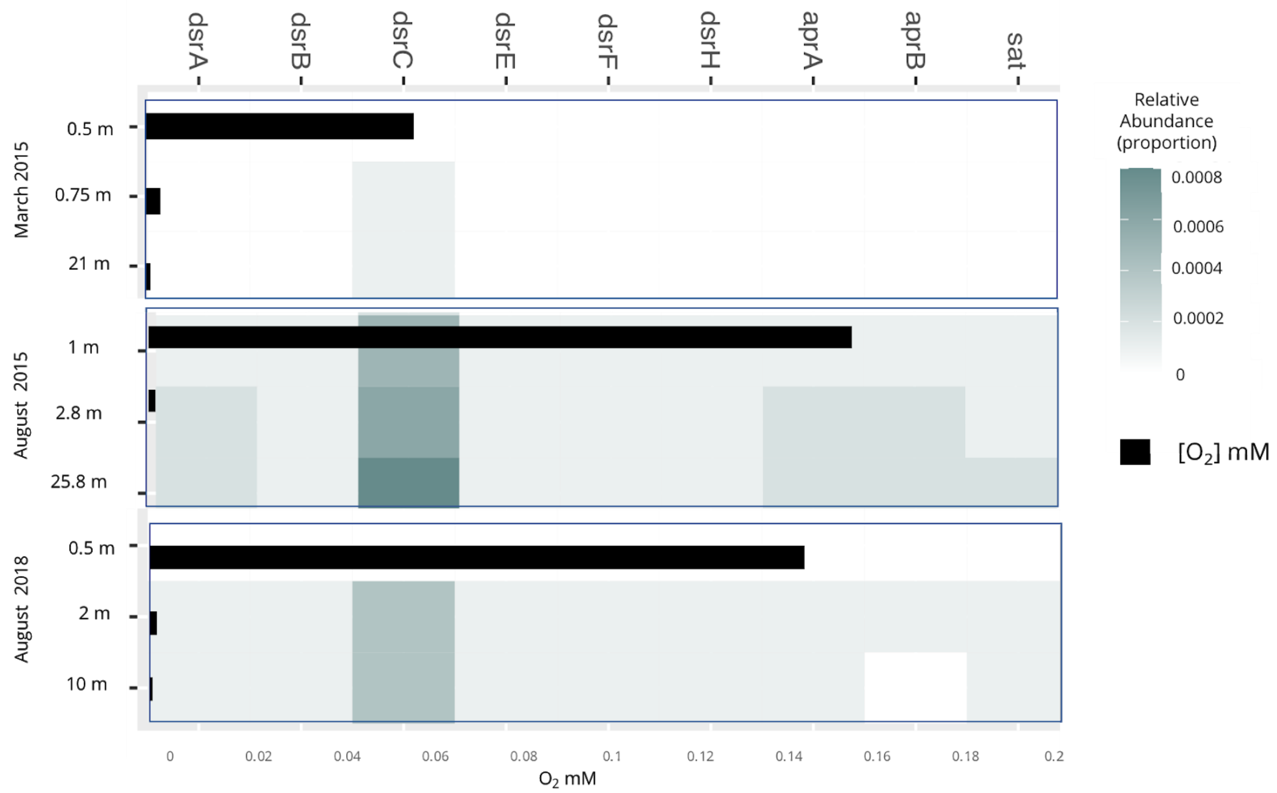

Figure S6 | Heat map of rDSR pathway gene relative abundances (dsrABCEFH, aprAB, sat) in March 2015, August 2015 and August 2018 through depth and overlaid oxygen concentrations (mM) (black bars) at corresponding depths.
