# Supplementary material for: O2 partitioning of sulfur oxidizing bacteria drives acidity and thiosulfate distributions in mining waters
Source: Nat Commun. 2023 Apr 10;14:2006. doi: 10.1038/s41467-023-37426-8 (PMC10086054; doi:10.1038/s41467-023-37426-8)
Supplement: Supplementary file 2 — Description of Additional Supplementary Files [file 41467_2023_37426_MOESM2_ESM.pdf]

## Description of Additional Supplementary Files:

**Supplementary Dataset 1:** 16SrRNA amplicons for mining waters (n=30) with Shannon's Diversity Index, Pielou's Eveness (J), richness and total sequences.

**Supplementary Dataset 2:** Physiochemical conditions across time (2015-2018) (21 sampling campaigns, n=53) of the Northern Ontario tailings impoundment water (temperature, pH, conductivity. oxidation-reduction potential (ORP), O2 % and [O2]. NA indicates that data is not available.

**Supplementary Dataset 3:** Sulfur speciation (n=53) (mmol/L) across time (2015-2018) of the tailings impoundment waters (TotS0.2µm,TotS0.45µm, TotSUF, S-SO42-, S-SO32-, S-S2O32-, S-S3 O62- (where available), S-S4 O62- (where available) ΣH2S and Sreact (defined as TotS0.45µm - S-SO42-).

**Supplementary Dataset 4:** Mine tailing impoundment aquatic microbial community composition within three statistical clusters 1) 2015 (n=8 2) 2016/2017 (n= 3 and 13) 2018 (n= 9) as determined through non-metric dimensional scaling (NMDS) and Curtis-Bray clustering analyses. Bold genera text denotes chemolithautotrophic sulfur oxidizing/disproportionating organisms.

**Supplementary Dataset 5:** Community level relative abundances of sulfur cycling nitrogen cycling, hydrogen cycling, carbon fixation, photosynthetic and aeronic respiration genes over time (2015-2018) in tailing impoundment waters.

**Supplementary Dataset 6:** Nitrogen speciation (N-NO3-, N-NO2- and N-NH4+ (unfiltered; mmol/L)), and total dissolved organic carbon (DOC) (0.45µm,; mmol/L) across time (2015-2018) of the tailings impoundment waters. All samples are shown as the average ± standard deviation.

**Supplementary Dataset 7:** Halothiobacillus sp. and Thiobacillus spp. RNA activities of August 2018 Tailings Impoundment waters at 0.5, 2.5 and 10 m depth
